# Efficacy of *Trichoderma longibrachiatum* SC5 Fermentation Filtrate in Inhibiting the *Sclerotinia sclerotiorum* Growth and Development in Sunflower

**DOI:** 10.3390/ijms26010201

**Published:** 2024-12-29

**Authors:** Enchen Li, Na Zhu, Shuwu Zhang, Bingliang Xu, Lilong Liu, Aiqin Zhang

**Affiliations:** 1 Gansu Provincial Biocontrol Engineering Laboratory of Crop Diseases and Pests, College of Plant Protection, Gansu Agricultural University, Lanzhou 730070, China; liec188@163.com (E.L.); 15693131025@163.com (N.Z.); 2 State Key Laboratory of Arid Land Crop Science, Gansu Agricultural University, Lanzhou 730070, China; 3 Institute of Animal Husbandry, Pasture and Green Agriculture, Gansu Academy of Agricultural Sciences, Lanzhou 730070, China; liull@gsagr.cn (L.L.); 2009aiqinhai@163.com (A.Z.); 4 Institute of Wheat Research, Gansu Academy of Agricultural Sciences, Lanzhou 730070, China

**Keywords:** *Trichoderma*, secondary metabolites, *Sclerotinia sclerotiorum*, growth and development inhibition, sclerotia production inhibition, species identification

## Abstract

*Sclerotinia sclerotiorum* is a destructive pathogen responsible for sunflower sclerotinia rot, resulting in substantial yield and economic losses worldwide. *Trichoderma* species have demonstrated the capacity to inhibit plant pathogen growth through the production of secondary metabolites. However, there are fewer recent studies focusing on the application of *Trichoderma* metabolites in inhibiting *S. sclerotiorum* growth and development and controlling sunflower sclerotinia rot disease. Our results showed that five *Trichoderma* strains (SC5, T6, TN, P6, and TS3) exhibited mycelial growth inhibition higher than 60% in dual culture assays out of the 11 tested strains. The *Trichoderma* SC5 fermentation filtrate exhibited superior efficacy compared to other strains, achieving a 94.65% inhibition rate of mycelial growth on *S. sclerotiorum*, 96% inhibition of myceliogenic germination of sclerotia, and 81.05% reduction in the oxalic acid content of *S. sclerotiorum*, while significantly increasing the cell membrane permeability. In addition, the *Trichoderma* SC5 fermentation filtrate significantly decreased the activities of polygalacturonase and pectin methyl-galacturonic enzymes and even caused *S. sclerotiorum* hyphae to swell, branch, twist, lyse, and inhibited the production and development of sclerotia. Moreover, the *Trichoderma* SC5 fermentation filtrate downregulated genes expression that associated with the growth and infection of *S. sclerotiorum*. The control efficacies of the protective and curative activities of the *Trichoderma* SC5 fermentation filtrate were 95.45% and 75.36%, respectively, on detached sunflower leaves at a concentration of 8 mg/mL. Finally, the *Trichoderma* SC5 was identified as *Trichoderma longibrachiatum* through morphological and phylogenetic analysis. Our research indicates that the *T. longibrachiatum* SC5 can be considered a promising biological control candidate against *S. sclerotiorum* and controlling the sunflower sclerotinia rot disease, both in vitro and in vivo.

## 1. Introduction

Sunflower (*Helianthus annuus* L.), the world’s fourth most widely cultivated oilseed crop, is recognized as a key source of premium edible oil for human consumption, biodiesel, and various industrial uses [1,2], with an expected annual production of 36 million metric tons [3]. Currently, sunflower diseases, including basal stem rot, head rot, and mid-stalk rot, caused by *Sclerotinia sclerotiorum*, pose a major threat to sunflower production in multiple regions of China [4]. The life cycle of *S. sclerotiorum* encompasses several critical stages, such as vegetative expansion, infection, sclerotia formation, myceliogenic and carpogenic germination of sclerotia, and apothecium development. The pathogen survives in the soil in the form of sclerotia, allowing it to withstand unfavorable climatic conditions. Sclerotia are aggregate of filamentous hyphae, formation of pigmented, hardened by the thin rind layer, and also containing nutrient reserve materials [5]. The production of multi-hyphal sclerotia is essential for the long-term survival of *S. sclerotiorum*, with these structures acting directly as infectious propagules [6]. *Sclerotinia sclerotiorum* employs cell wall degrading enzymes (CWDEs) and oxalic acid (OA) as its main virulence factors to break down host cell structures and extract nutrients [7]. Researches have shown that CWDEs secreted by *S. sclerotiorum* can effectively break down plant tissues, degrade cell wall components, promote pathogen invasion [8,9], and even secrete OA, a versatile virulence factor that modulates host defenses and establishes an acidic milieu, enabling optimal and efficient functioning of fungal CWDEs [10,11]. The complexity of its pathogenic mechanisms is reflected in the involvement of various genes and secretory proteins. Number of studies have reported that the *S. sclerotiorum* genome contains coding sequences for over 600 secreted proteins, with 70 potential prediction and identification effectors [12,13]. For instance, the Cu/Zn superoxide dismutase genes (*SsSOD1*, STNG_00699) play the crucial role in scavenging reactive oxygen species during host-pathogen interaction and are the primary virulence factors of *S. sclerotiorum* [14]. The potential effector integrin-like protein SsITL from *S. sclerotiorum* can interfere with the plant salicylic acid signaling pathway in the early stages of infection, thereby suppressing the host immune response [15].

Biological control agents (BCAs) of antagonistic microorganisms provide an appealing alternative for safeguarding crops against pathogens without the detrimental consequences of chemical fungicides. Several fungi, including *Coniothyrium minitans*, *Trichoderma* spp., and *Aspergillus* spp., have been explored as potential BCAs against *S. sclerotiorum* [16,17]. Various *Trichoderma* species have been utilized as BCAs to combat plant diseases triggered by diverse soil-borne pathogens, such as *Rhizoctonia solani*, *Fusarium verticillioides*, *F. oxysporum*, and *S. sclerotiorum* [18,19,20,21,22]. *Trichoderma harzianum* 303/02 exhibited mycoparasitic potential by growing alongside hyphae of *S. sclerotiorum* and coiling firmly around them, while simultaneously multiplying abundantly on the surface of sclerotia and apothecia, eventually forming dense mycelium that penetrated the interior surface of these structures [23]. Furthermore, some *Trichoderma* strains enhance plant growth and induce the upregulation of defense-related genes expression, which in turn activate plant defense pathways and exhibit biocontrol effectiveness against *S. sclerotiorum* [24,25]. Research on the biochemical and molecular mechanisms of *Trichoderma* spp. metabolites against *S. sclerotiorum* are currently scarce. Notably, *Trichoderma* species are well known for their production of diverse bioactive secondary metabolites, such as gliotoxin, peptaibols, gliovirin, terpenes, polyketides, and pyrones, which have shown considerable biological efficacy in combating numerous plant pathogenic fungi, bacteria, and nematodes [26,27,28]. A prior study indicated that volatile compounds produced by *Trichoderma* spp. can significantly suppress mycelial growth, sclerotia formation, and germination [29]. Nevertheless, few studies have addressed how *Trichoderma* fermentation filtrate influences the morphological development and physiological–biochemical properties of *S. sclerotiorum*.

The present study aims to (1) screen the *Trichoderma* strains for inhibiting mycelial growth and sclerotia production of *S. sclerotiorum*; (2) evaluate the effects of *Trichoderma* fermentation filtrate on the morphological and physiological characteristics of *S. sclerotiorum*; (3) identify the candidate *Trichoderma* strain and analyze the effects of its fermentation filtrate on the growth and infection-related genes of *S. sclerotiorum* expression; and (4) test the protective and curative efficacy of the candidate *Trichoderma* strain fermentation filtrate in controlling sclerotinia rot disease on the detached leaves of sunflower.

## 2. Results

### 2.1. Trichoderma Strains and S. sclerotiorum Dual Culture Test

**Figure 1:**
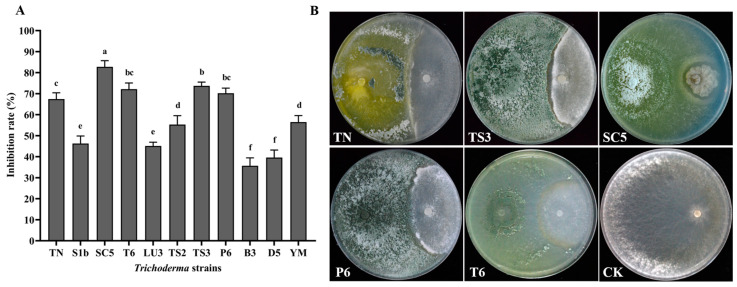
Inhibitory effect (**A**) and antagonistic activity (**B**) of *Trichoderma* strains on *S. sclerotiorum* at 7 days after incubation, where CK is the control of *S. sclerotiorum* without confrontation. Means with different letters are significantly different at *p* < 0.05 according to Duncan’s multiple range test.

*Trichoderma* strains exhibited significant inhibitory potential against *S. sclerotiorum* in dual culture plates. The strain of SC5 showed the highest inhibition rate with 82.75%, while the B3 strain showed the lowest inhibition rate with 35.68% among all the *Trichoderma* strains. In addition, the strains of T6, TN, P6, and TS3 presented a higher inhibitory potential of more than 60% (Figure 1A). Antagonistic assessment results showed that the five *Trichoderma* strains (SC5, T6, TN, P6, and TS3) had potential activity against *S. sclerotiorum*. These five *Trichoderma* strains rapidly colonized the available nutrients and space necessary for development, effectively inhibiting the mycelial expansion of *S. sclerotiorum*. The strains of SC5 and TS3 produced a clear inhibition zone ahead of the *S. sclerotiorum* colony. In contrast, the SC5 and T6 strains can cause the mycelia of *S. sclerotiorum* discoloration and turn brown (Figure 1B).

### 2.2. Effect of Trichoderma Strains Fermentation Filtrate on Mycelial Growth, Hyphal Morphology, and Sclerotia Formation of S. sclerotiorum

**Figure 2:**
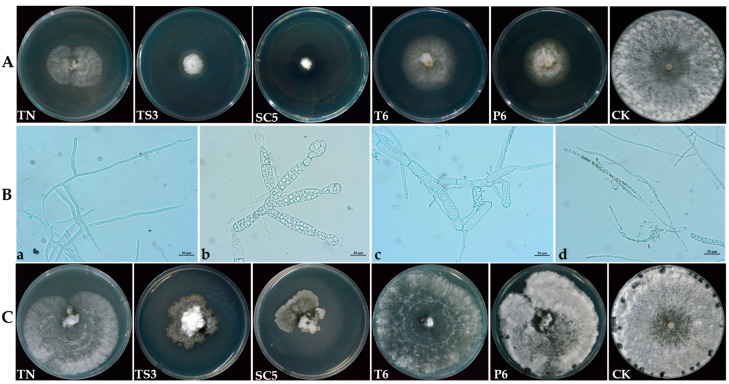
The inhibitory effect of different *Trichoderma* spp. fermentation filtrate on *S. sclerotiorum*. (**A**): Effects of different *Trichoderma* spp. fermentation filtrate on the mycelial growth of *S. sclerotiorum* on PDA plates at 5 days. (**B**): Effect of SC5 fermentation filtrate on hyphal morphology of *S. sclerotiorum* at 5 days after inoculation. (**a**) Healthy hyphae of *S. sclerotiorum*; (**b**) hyphae to branch; (**c**) treated hyphae with twisted; (**d**) hyphae lysis. (**C**): The effect of *Trichoderma* spp. fermentation filtrate on the mycelial growth and sclerotial formation of *S. sclerotiorum* at 20 days after incubation.

The colony diameter and sclerotia count of *S. sclerotiorum* were significantly decreased after treatment with the fermentation filtrate of different *Trichoderma* strains. Colonies grown on PDA media containing 10% (*v*/*v*) fermentation filtrate were significantly smaller than the control colony (Figure 2A), the inhibition rates of *Trichoderma* TN, TS3, SC5, T6, and P6 fermentation filtrate against *S. sclerotiorum* were 69.58%, 83.01%, 94.65%, 58.99%, and 74.59% at 5 days after inoculation, respectively (Table 1). Microscopic examination showed that the SC5 fermentation filtrate had a significant impact on the hyphal morphology of *S. sclerotiorum*. The hyphae without SC5 fermentation filtrate treatment were uniseriate, uniform, and with smooth surfaces (Figure 2B-a). Following treatment with SC5 fermentation filtrate, *S. sclerotiorum* hyphae became swollen, the hyphal branching of the apical increased, and the intervals between the septa were shortened compared to the control (Figure 2B-b,c). Moreover, the hyphae of *S. sclerotiorum* were twisted and lysed after treatment with SC5 fermentation filtrate (Figure 2B-c,d).

Compared to the control, *S. sclerotiorum* demonstrated slower and sparser mycelial growth, with inhibited sclerotia formation on PDA supplemented with various *Trichoderma* fermentation filtrate at 20 days post-inoculation. Moreover, TS3 and SC5 fermentation filtrate caused the mycelia of *S. sclerotiorum* to turn brown (Figure 2C). No sclerotia formation was observed after treatment with the fermentation filtrate of TN, TS3, and SC5 strains. The number of sclerotia were 9.0 ± 1.0 and 15.3 ± 2.1 per plate after treatment with T6 and P6 fermentation filtrate, respectively, which were significantly lower than that of the control (Table 1). Meanwhile, the dry weight of sclerotia showed no significant difference between the P6 fermentation filtrate treatment and the control. The dry weight of sclerotia was 0.0577 g after treatment with T6 fermentation filtrate, which was significantly lower than the control (Table 1).

### 2.3. Effect of Trichoderma Strains Fermentation Filtrate on Sclerotia Myceliogenic Germination

**Figure 3:**
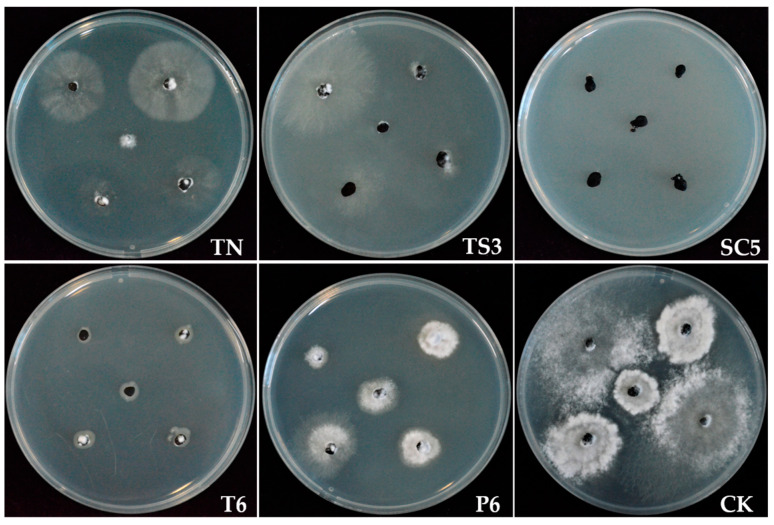
Effect of different *Trichoderma* spp. fermentation filtrate on myceliogenic germination of sclerotia on PDA at 5 days.

Compared with the control, the inhibition rates for the myceliogenic germination of sclerotia were 96%, 59%, 68%, 76%, and 15% after treatment with SC5, TN, TS3, T6, and P6 strains fermentation filtrate, respectively. Meanwhile, the mycelial growth was slower and sparser than the control after treatment with TN, TS3, and T6 fermentation filtrate. The growth rate of sclerotia germination mycelia was slower than that of the control after treatment with the P6 fermentation filtrate (Figure 3). However, the sclerotia germinated, became thicker and denser, and formed white mycelium after being treated with sterile water. Sclerotia treated with SC5 fermentation filtrate hardly germinated, and hyphal growth was limited to the sclerotial surface.

### 2.4. Effect of Trichoderma Strains Fermentation Filtrate on Oxalic Acid Content and Cell Membrane Permeability of S. sclerotiorum

**Figure 4:**
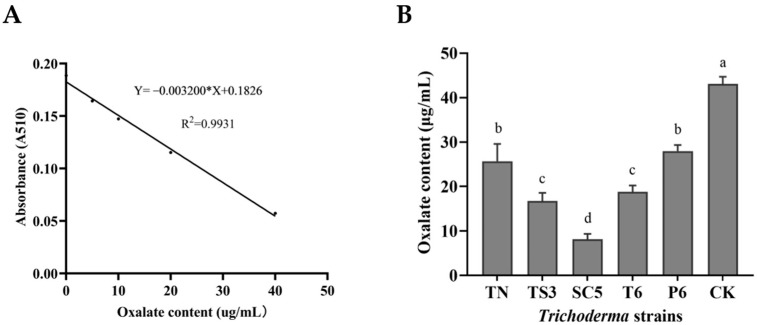
Standard curve for calculating oxalic acid content (**A**) and oxalic acid content of *S. sclerotiorum* after being treated with five *Trichoderma* strains fermentation filtrate (**B**). Bars represent the standard deviations of the means. According to Duncan’s multiple range test, there are significant differences (*p* < 0.05) between the mean values in columns labeled with the different letters.

Oxalic acid levels were determined through standard curve analysis (Figure 4A). The oxalic acid content of *S. sclerotiorum* treated with the fermentation filtrate of five *Trichoderma* strains was significantly lower than that of the control. The oxalic acid contents of *S. sclerotiorum* were decreased by 40.36%, 61.11%, 81.05%, 56.31%, and 35.04% after being treated with the fermentation filtrate of TN, TS3, SC5, T6, and P6, respectively, compared to the control (Figure 4B).

**Figure 5:**
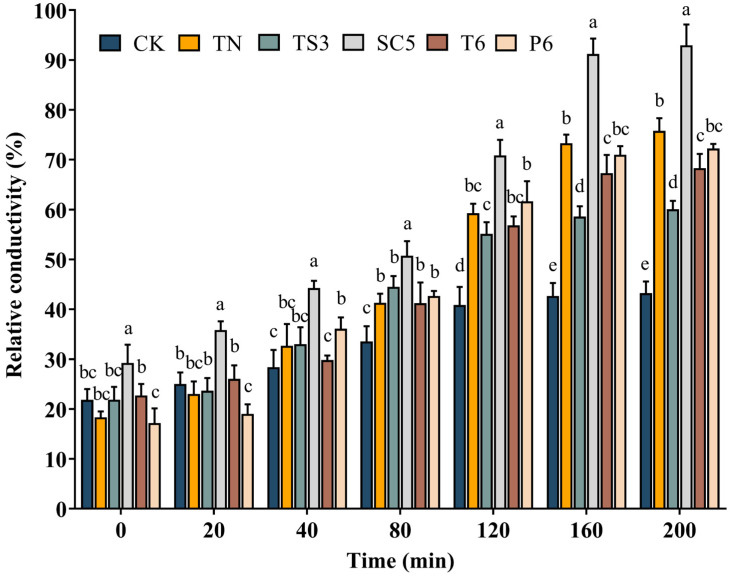
Effect of *Trichoderma* spp. fermentation filtrate on the relative conductivity of *S. sclerotiorum* mycelium. Bars represent the standard deviations of the means. Means labeled with the different letters for each time period indicate significant differences (*p* < 0.05) according to Duncan’s multiple range test.

The relative conductivity of *S. sclerotiorum* mycelium was increased with the treatment time and finally stabilized after being treated and untreated with different *Trichoderma* spp. fermentation filtrate (Figure 5). However, throughout the entire treatment period, the relative conductivity of all treatment groups was consistently higher than the control group. The *Trichoderma* SC5 fermentation filtrate presented the most pronounced impact on *S. sclerotiorum* cell membrane permeability among the five tested *Trichoderma* strains. Compared with the control, the relative conductivity of *S. sclerotiorum* mycelium was increased by 49.72% after treatment with the SC5 fermentation filtrate for 200 min. These findings indicate that *Trichoderma* spp. fermentation filtrate potentially compromise cell membrane integrity, resulting in enhanced electrolyte leakage from *S. sclerotiorum* mycelium.

### 2.5. Effect of Trichoderma Strains Fermentation Filtrate on Polygalacturonase (PG) and Pectin Methyl-Galacturonase (PMG) Activitives of S. sclerotiorum

**Figure 6:**
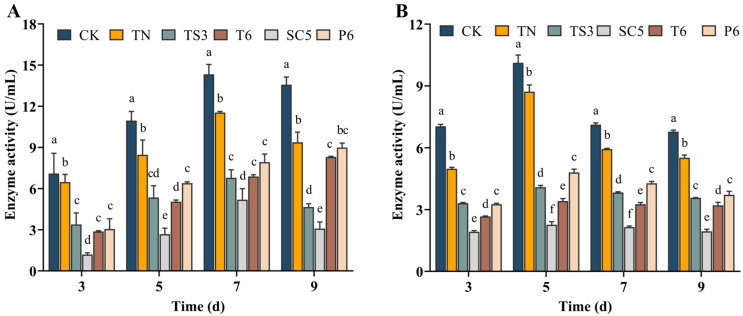
Effect of *Trichoderma* spp. fermentation filtrate on the cell wall degrading enzymes (**A**) PG and (**B**) PMG activities of *S. sclerotiorum*. Bars represent the standard deviations of the means. Means labeled with the different letters for each time period indicate significant differences (*p* < 0.05) according to Duncan’s multiple range test.

Both PG and PMG activities in all treatments and control were increased initially and then decreased over time. Nevertheless, the PG and PMG activities of *S. sclerotiorum* were significantly diminished compared to the control group during different culture periods (*p* < 0.05) after treatment with *Trichoderma* spp. fermentation filtrate. The PG enzyme activities of *S. sclerotiorum* were 11.54 ± 0.08, 6.79 ± 0.58, 5.19 ± 0.80, 6.89 ± 0.12, and 7.93 ± 0.59 U/mL after treatment with the TN, TS3, SC5, T6, and P6 fermentation filtrate, respectively, which were significantly lower than the control (14.33 ± 0.71 U/mL) at day 7 (Figure 6A). However, the PMG activities were 8.73 ± 0.32, 4.09 ± 0.09, 2.27 ± 0.15, 3.41 ± 0.12, and 4.80 ± 0.16 U/mL after treatment with the TN, TS3, SC5, T6, and P6 fermentation filtrate at day 5, respectively, which were significantly lower than the control value of 10.12 ± 0.16 U/mL (Figure 6B). Compared with other strains, the strain of SC5 fermentation filtrate had the highest inhibition effect on PG and PMG enzyme activities at different days, respectively (Figure 6).

### 2.6. Effect of Trichoderma SC5 Fermentation Filtrate on the Growth and Infection-Related Genes of S. sclerotiorum Expression

**Figure 7:**
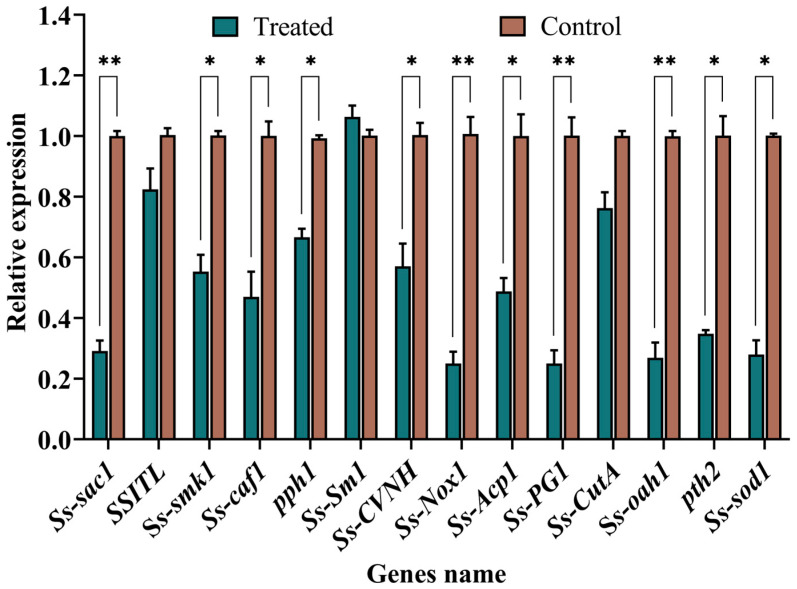
Relative expression levels of 14 genes of *S. sclerotiorum* after treatment with the *Trichoderma* SC5 fermentation filtrate at 5 days. Bars indicate the standard deviation, and asterisks (*) indicate the significant differences (one-way ANOVA): * *p* < 0.05 and ** *p* < 0.01.

Following a 5-day treatment with SC5 fermentation filtrate, the relative expression levels of *Ss-sac1*, *Ss*-*Smk1*, *Ss-Caf*, *Ss-Pph1*, *Ss-Sm1*, *Ss-CVNH*, and *Ss-Nox1*, which are genes related to mycelial growth and sclerotial production in *S. sclerotiorum*, were significantly reduced 3.44, 1.81, 2.13, 1.50, 1.75, and 4.00-folds relative to the control (Figure 7). In contrast, the expression levels of *Ss-Acp1*, *Ss-Pg1*, and *Ss-CutA* genes were downregulated 2.05, 4.00, and 1.31-folds among the genes for regulating the biosynthesis of *S. sclerotiorum* CWDEs, respectively. Moreover, the expression levels of genes related to the oxalic acid synthesis and signaling pathways of *S. sclerotiorum* were significantly inhibited (*p* < 0.05) after treatment with SC5 fermentation filtrate, and the expression levels of *Ss-oah1*, *Ss-pth2*, and *Ss-sod1* were down-regulated 3.72, 2.94 and 3.58-folds, respectively, compared to the control (Figure 7).

### 2.7. Protective and Curative Activities of Trichoderma SC5 Lyophilized Fermentation Filtrate

**Figure 8:**
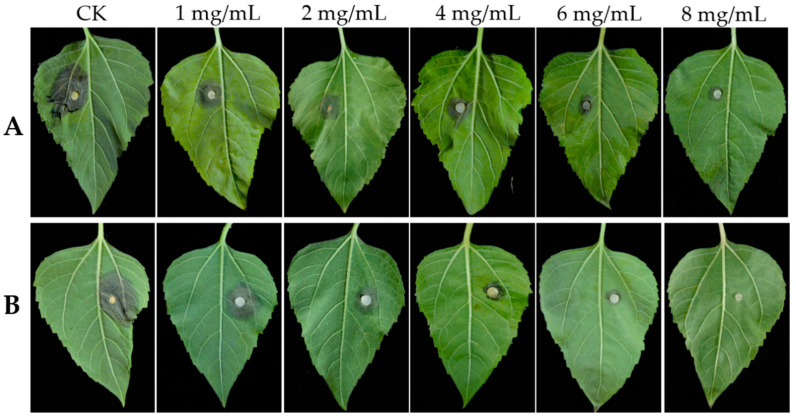
Curative (**A**) and protective activities (**B**) of *Trichoderma* SC5 fermentation filtrate on sunflower leaves after inoculation.

The lyophilized fermentation filtrate of *Trichoderma* SC5 demonstrated both the protective and curative effects on sunflower leaves after inoculation, and the protective and curative effects were increased in a dose-dependent manner. In the protective activity assay, the control efficacies of the different SC5 fermentation filtrate dilutions ranged from 39.39 to 95.45%. The highest control efficacies were recorded by 79.17% and 95.45% after the application of SC5 fermentation filtrate at 6 and 8 mg/mL, respectively (Table 2, Figure 8B). In the curative activity assay, the control efficacies of the different SC5 fermentation filtrate dilutions ranged from 38.03 to 75.36%. The control efficacy was recorded by 75.36% at 8 mg/mL after the application of SC5 fermentation filtrate (Table 2, Figure 8A). In summary, the protective activity of SC5 fermentation filtrate on sunflower leaves was higher than the curative activity with the same concentration.

### 2.8. Trichoderma SC5 Strain Identification

**Figure 9:**
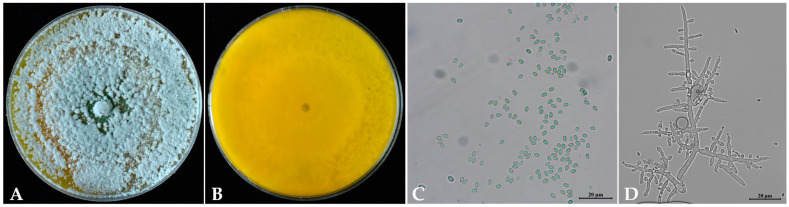
Morphological characteristics of *Trichoderma* SC5 that cultured on PDA medium. (**A**) front colony; (**B**) reverse colony; (**C**) conidia; (**D**) conidiophores. Photographs were taken at 5 days post-inoculation.

**Figure 10:**
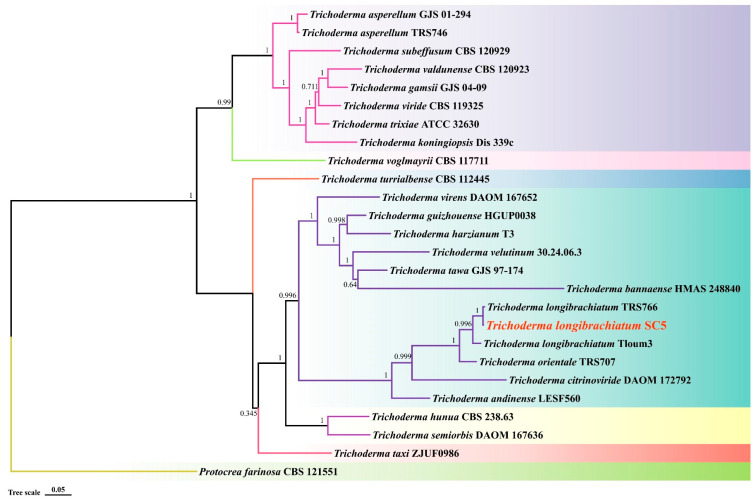
Multi-locus phylogram of the present *Trichoderma* SC5 (red words) based on the combination of ITS, *EF-1α1,* and *RPB2* genes sequence. *Protocrea farinose* (CBS 121551) was selected as outgroup. Bootstrap values at the nodes are based on 2000 replicates. The colors represent clades shown on the concatenated phylogram of the three loci.

The colony of *Trichoderma* SC5 exhibits rapid growth on the PDA medium and displays a dense white aerial mycelium at the initial stages according to the morphological characteristics. Over time, the colony transitions in color, changing from yellow-green to green, with yellow pigmentation on the reverse side (Figure 9A,B). The conidiophores typically possess multiple branching levels, and branches bear a single phialide without further branching near the tip, while distal branches exhibit rebranching patterns (Figure 9D). The phialides are cylindrical to lageniform in shape, often swollen in the middle, and can be solitary or terminating in a single cell. They are frequently subtended by an intercalary phialide and may appear straight, hooked, or sinuous in shape. The conidia are smooth and exhibit variation in shape and size, ranging from ellipsoidal to oblong nearly (Figure 9C). The sizes of the conidia ranged from 2.7 to 3.5 × 5 to 7.6 μm (*n* = 100). Based on the phylogenetic analyses, the maximum likelihood resulted from a combined three-locus dataset of the internal transcribed spacer (ITS), the translation elongation factor-1-α (*EF1-α1*), and the RNA polymerase subunit B II (*RPB*2). Our findings demonstrated that the sequence of each gene of SC5 strain was 100% identical to *T. longibrachiatum*, which fell into a subclade with 100% bootstrap supports (Figure 10). Finally, the strain of *Trichoderma* SC5 was identified as *T. longibrachiatum* according to the morphological and multi-locus analysis.

## 3. Discussion

Currently, the main strategies to prevent and control sclerotinia rot disease that caused by *S. sclerotiorum* including cultural control and the application of synthetic fungicides. The crop rotation practice focuses on reducing soil sclerotia counts, which poses minimal harm to the environment. However, crop rotation measures may prove insufficient in many cases, especially when they involve high-value crops or highly specialized farms. Owing to the escalating public awareness regarding the polluting, residual, resistant, and phytotoxic effects associated with numerous synthetic fungicides, employing biotechnological approaches for sclerotinia rot disease management have emerged as a prominent area of research [30,31]. In this study, a total of eleven *Trichoderma* strains were evaluated for their efficacy against *S. sclerotiorum*. Five *Trichoderma* strains (namely SC5, TN, TS3, T6, and P6) exhibited higher growth inhibition of *S. sclerotiorum*. During the dual culture of SC5 and *S. sclerotiorum* period, a distinct inhibition zone was observed between the SC5 and *S. sclerotiorum*. Colonies connecting area that makes the *S. sclerotiorum* fail to grow may be attributed to the antifungal compounds produced by *Trichoderma* SC5 metabolites. Thus, analyzing the antifungal activity of these metabolites against pathogens can serve as a simple and effective screening tool for isolating potential biocontrol strains and further identifying their bioactive substances [32].

The use of bioactive secondary metabolites from microorganisms as substitutes for synthetic fungicides to control plant diseases has been identified as a promising, environment friendly, and consumer-friendly strategy [33]. Previous studies have reported that various enzymes and secondary metabolites secreted by *Trichoderma* exhibit antifungal activity. For instance, *T. harzianum* T23 effectively inhibits the germination of *Verticillium dahliae* conidia, *Phytophthora infestans* sporangia, and *S. sclerotiorum* sclerotia by producing viridiofungin A (VFA) [34]. Lytic enzymes, chitinase, and *β*-1,3-glucanase, produced by *T. erinaceum*, exhibit direct antagonistic effects on pathogen growth by degrading their cell walls [35]. In the current study, the metabolites from the fermentation filtrate of five *Trichoderma* strains exhibited significant antifungal activity, especially *T. longibrachiatum* SC5, which showed the strongest inhibitory effect on *S. sclerotiorum*. However, the fermentation filtrate of five *Trichoderma* strains exhibited different levels of antifungal activities against *S. sclerotiorum*, which were possibly influenced by the types of produced metabolites and lytic enzymes. Research has shown that the production of bioactive secondary metabolites depends on the strains and species of biocontrol fungi, and that the biochemical and metabolic characteristics of the same *Trichoderma* strain vary across different agro-ecosystems [36,37,38].

Choosing highly active microbial products is a crucial strategy for replacing traditional chemical fungicides, but they can only be efficiently applied by clarifying their molecular targets. The current research results showed that among the five *Trichoderma* strains, the antagonism of SC5 fermentation filtrate against *S. sclerotiorum* demonstrated the potential for biological control application. The inhibition rate of *S. sclerotiorum* mycelial growth by the SC5 fermentation filtrate reached 94.65%, which is comparable to the reported effects of the commercial fungicide Frowncide 500 SC^®^ [39], and also the SC5 fermentation filtrate inhibited the production and germination of sclerotia. Research indicates that methanolic and ethyl acetate extracts from two *T. asperelloides* strains significantly inhibit *S. sclerotiorum* mycelial growth, sclerotia development, and germination [39]. Volatile and non-volatile metabolites produced by *Bacillus cereus* (SC-1 and P-1) and *B. subtilis* (W-67) significantly inhibited the mycelial growth of *S. sclerotiorum* and completely suppressed sclerotia germination [40]. In this study, the SC5 fermentation filtrate showed a more significant effect in inhibiting the production and germination of sclerotia completely.

In current study, we found that the *Trichoderma* SC5 fermentation filtrate can lead to the intracellular electrolyte leakage of *S. sclerotiorum* mycelium increased and the mycelium structure damaged after observed by light microscope. Similarly, *S. sclerotiorum* hyphal tips showed twisting, enhanced apical branching, cell membrane damage, and increased electrolyte leakage [41,42] after treatment with fluazinam and fludioxonil. *Purpureocillium lilacinum* culture filtrate suppressed the mycelial growth and sclerotia development of *S. sclerotiorum* by leading to the increased protoplast leakage of its hyphae [43]. Previous studies have demonstrated that *S. sclerotiorum* can secrete some CWDEs during the infection process that aim to destroy the plant cell wall. Furthermore, the breakdown products of pectinases act as nutritional resources, facilitating the progression of *S. sclerotiorum* infection [6]. In this study, we found that treatment with the SC5 fermentation filtrate significantly reduced the PG and PMG activities of *S. sclerotiorum*, which indicate that the reduction activities of PG and PMG by the fermentation filtrate may affect the pathogenicity of *S. sclerotiorum*.

In addition to CWDEs, OA is also a major pathogenic factor of *S. sclerotiorum* that has been demonstrated to inhibit the host’s reactive oxygen species burst, disrupt the function of host guard cells, alter the redox status of the host cells, and even induce programmed cell death in host cell [10,44,45]. In this study, OA content significantly decreased after treatment with different *Trichoderma* spp. fermentation filtrate, with SC5 treatment resulting in the lowest OA content. This indicates that the *Trichoderma* strain SC5 fermentation filtrate could inhibit the production of OA or degrade OA to reduce the pathogenicity of *S. sclerotiorum*. A previous report indicates that the culture filtrate of *P. lilacinum* significantly reduces the oxalic acid production by *S. sclerotiorum* and the severity of soybean white mold [43]. The mode of action of biocontrol strain cultures is consistent with the results of our study.

To elucidate the molecular mechanism by which SC5 fermentation filtrate inhibits *S. sclerotiorum*, the genes related to *S. sclerotiorum* growth and infection were analyzed by transcriptional analysis. After SC5 fermentation filtrate treatment, the relative expression levels of the growth and infection-related genes of *S. sclerotiorum* were significantly down-regulated in comparison to the control. Our results suggest that these genes are the primary targets of SC5 fermentation filtrate, which makes SC5 an effective antifungal agent against *S. sclerotiorum*. However, *Ss-sm1* gene expression increased, which merits further study.

The secondary metabolites produced by *Trichoderma* spp. confer a selective advantage through mechanisms including competition, mycoparasitism, symbiosis, mineral transport, growth promotion, and sensing [27,46]. In addition to directly acting on pathogens, some of these substances can also induce the up-regulation of *PR* genes and other defense-related genes to trigger the plant’s defense response [47]. Furthermore, Hermosa et al. [48] demonstrated that the interaction between plants and biocontrol fungi is a complex cross-talk, with *Trichoderma*-induced plant resistance being associated with the SA and JA/ET pathways. Our findings revealed that the lyophilized fermentation filtrate derived from SC5 exhibits both protective and curative activity on sunflower leaves after inoculation with *S. sclerotiorum*, which indicate the potential for development as a biocontrol agent. Nevertheless, the field effectiveness of fermentation filtrate in controlling sunflower sclerotinia rot disease needs further study in the future.

## 4. Conclusions

The *Trichoderma* SC5 fermentation filtrate showed the highest inhibitory effects on the growth, development, and pathogenicity of *S. sclerotiorum*. This effect is primarily achieved by suppressing mycelial growth, sclerotia formation, and germination, disrupting mycelial structure, reducing pathogenic enzyme activity and oxalic acid secretion, and even downregulating the expression levels of certain growth- and infection-related genes. Finally, the SC5 strain was identified as *T. longibrachiatum*, based on its morphological characteristics and multi-locus sequence analysis. The newly identified *Trichoderma longibrachiatum* strain demonstrated great potential in significantly reducing the incidence and severity of sunflower sclerotinia rot. These findings highlight the emerging trend of using natural products to control plant diseases, contributing to the sustainable development of agricultural production.

## 5. Materials and Methods

### 5.1. Pathogen Isolation and Purification

*Trichoderma* strains (T6, S1b, LU3, TN, D5, P6, B3, TS2, TS3, T-Ym, and SC5) were sourced from the Plant Virology and Molecular Biology Laboratory, Gansu Agricultural University, China. The *S. sclerotiorum* was isolated from the main sunflower planting bases in Minqin County, Gansu Province, China, and cultured on potato dextrose agar (PDA) medium in Petri dishes at 25 °C. Following 30 days of incubation, sclerotia were harvested from the *S. sclerotiorum* colony, immersed in 75% alcohol for 20 s, and then subjected to 0.5% sodium hypochlorite solution for 5 min. The sterilized sclerotia were subsequently washed five times with sterile water, air-dried, and preserved at 4 °C for future experimental use.

### 5.2. Antagonistic Activity of Trichoderma Strains Against S. sclerotiorum

Eleven *Trichoderma* strains were tested for antagonistic activity against *S. sclerotiorum* on PDA media by using the dual-culture method. Mycelial discs (5 mm in diameter) from the colony edges of 3-day-old cultures of *S. sclerotiorum* and *Trichoderma* strains were placed equidistantly on fresh PDA plates. The *S. sclerotiorum* alone inoculation was used as a control. All plates were incubated at 25 °C, with three replicates for each strain. The colony diameters of *S. sclerotiorum* in the treatment and control groups were measured at 7 days post-inoculation, with the diameter of the initial inoculation plug excluded. The radial growth inhibition percentages of *S. sclerotiorum* mycelium by *Trichoderma* strains were determined according to the following formula: radial growth inhibition rate (%) = [(R1 − R2)/R1] × 100 [49], where R1 and R2 were the mycelial radial growth of *S. sclerotiorum* in the control and treatment, respectively. The experiment was conducted thrice.

### 5.3. Trichoderma Strains Fermentation Filtrate Preparation

The *Trichoderma* strains SC5, T6, TN, P6, and TS3 were cultured on PDA plates at 25 °C for 5 days. According to the method of Zhang et al. [50], the conidia suspension of *Trichoderma* strains was prepared and adjusted to a concentration of 1 × 10^7^ conidia per milliliter. The conidia suspension (1 mL) was combined with 90 mL of potato dextrose broth (PDB) in a 250 mL Erlenmeyer flask and fermented on a shaker at 28 °C for 9 days. Upon completion of fermentation, centrifugation was performed at 4 °C and 10,000 rpm for 20 min to separate and remove the *Trichoderma* mycelium. The supernatant was repeatedly filtered twice with a Millipore syringe filter (0.22 μm), and then the obtained fermentation filtrates were stored at 4 °C.

### 5.4. Impact of Trichoderma Strains Fermentation Filtrate on S. sclerotiorum Mycelial Growth and Hyphal Morphology

The effects of *Trichoderma* fermentation filtrate on the radial growth of *S. sclerotiorum* mycelium were evaluated on PDA plates. For this step, 90 mL of PDA medium was combined with 10 mL of fermentation filtrate, with a mixture of sterile water and PDA serving as the control treatment. A 3-day-old mycelial disc (5 mm in diameter) of *S. sclerotiorum* was positioned at the center of plates with different treatments and incubated at 25 °C. After 5 days of incubation, the colony diameter was measured in two perpendicular directions, and the average was calculated finally. The growth inhibition (GI) percentage was determined using the following formula: GI (%) = [(d_c_ − d_t_)/d_c_] × 100 [51], where d_c_ denotes the *S. sclerotiorum* colony diameter in the absence of *Trichoderma* fermentation filtrate, and d_t_ represents the *S. sclerotiorum* colony diameter on PDA medium containing *Trichoderma* fermentation filtrate. After 5 days of culture, the morphology of *S. sclerotiorum* hyphae in plates containing fermentation filtrate (the highest inhibition rate) and plates without any fermentation filtrate were observed by the Nikon DS-Ri2 microscope. The number and dry weight of sclerotia produced on each plate were assessed after 20 days. Each treatment included three plates, and the experiment was conducted in triplicate.

### 5.5. Effect of Trichoderma Strains Fermentation Filtrate on Sclerotial Myceliogenic Germination

The impact of fermentation filtrate from *Trichoderma* strains (TN, TS3, SC5, T6, and P6) on sclerotia myceliogenic germination was investigated following the methodology outlined by Elsherbiny et al. [43]. Briefly, sterilized sclerotia with similar size and weight were soaked in 50 mL fermentation filtrate for 30 min. The sclerotia treated with sterile water served as the control. After drying, the treated sclerotia were cultured on PDA at 25 °C. Each treatment utilized 100 sclerotia. Myceliogenic germination of sclerotia was observed and recorded at 7 days post-inoculation. The inhibition percentage of myceliogenic germination of sclerotia by various *Trichoderma* fermentation filtrate was determined according to the following formula: inhibition of germination rate (%) = 100 − [(quantity of sclerotia with myceliogenic germination in treatment/quantity of sclerotia with myceliogenic germination in control) × 100].

### 5.6. Effect of Trichoderma Strains Fermentation Filtrate on Cell Membrane Permeability of S. sclerotiorum

The impact of *Trichoderma* strains fermentation filtrate on *S. sclerotiorum* cell membrane permeability was evaluated using an improved method based on Elsherbiny et al. [43]. Three mycelial discs (d = 5 mm) were collected from the margin of a 3-day-old *S. sclerotiorum* colony and inoculated into a 250 mL Erlenmeyer flask with 90 mL of PDB. The flask was incubated at 160 rpm and 25 °C in a shaking incubator for 48 h. Following incubation, fermentation filtrate was added to the flasks at a 10% (*v*/*v*, i.e., a mixture of 90 mL PDB medium and 10 mL *Trichoderma* strain fermentation filtrate) concentration. The same volume of sterile water was added as the control. After continuing incubation with shaking under the same conditions for 36 h, the mycelium was collected by filtering through three layers of filter paper and washed three times with sterile water. Excess water from the mycelium was removed by vacuum filtration for 20 min, followed by the transfer of 0.5 g of mycelium from each treatment into 20 mL of double-distilled water. To assess cell membrane permeability and cellular content release, the conductivity of mixture double-distilled water was measured at 0, 20, 40, 80, 120, 160, and 200 min intervals using a DDS-307A digital conductivity meter (INESA, Shanghai, China). After 200 min, the mycelial suspensions were boiled for 5 min to measure the final conductivity. Mycelial relative conductivity was computed using the following equation: relative conductivity (%) = (conductivity/final conductivity) × 100. Each treatment had three replicates, and the experiment was repeated three times.

### 5.7. Effect of Trichoderma Strains Fermentation Filtrate on Oxalic Acid Content of S. sclerotiorum

The oxalic acid content was determined according to the method described by Hou et al. [41]. Five mycelial discs (d = 5 mm) were taken from the edge of a 3-day-old *S. sclerotiorum* colony and inoculated into a flask containing 100 mL of PDB, with a final concentration of 5% (*v*/*v*) *Trichoderma* fermentation filtrate in the PDB. An equal volume of sterile water was added to the PDB as the control. After shaking the flask at 170 rpm and 25 °C for 5 days, and then centrifuging at 1500 rpm for 10 min was carried out to remove the pellet and retain the supernatant. The absorbance of the supernatant was measured at 510 nm using an ELx800 microplate reader (BioTek Instruments, Inc., Winooski, VT, USA). The OA content in the supernatant from different treatments was calculated based on the standard curve. Each treatment was repeated three times, and the experiment was conducted in triplicate.

### 5.8. Effect of Trichoderma Strains Fermentation Filtrate on CWDE Activity of S. sclerotiorum

Five mycelial discs (d = 5 mm) of *S. sclerotiorum* cultured on PDA for 3 days were transferred to the flask containing 90 mL of PDB amended with *Trichoderma* strain fermentation filtrate at the ultimate concentration of 5% (*v*/*v*). The same volume of sterile water was added to the PDB culture medium as the control. The flask was incubated on a shaker at 25 °C and 180 rpm for 3, 5, 7, and 9 days, and then centrifuged at 8500 rpm for 30 min at 4 °C to remove the pellet and retain the supernatant. The activities of PG and PMG in *S. sclerotiorum* culture supernatants were determined using the 3,5-dinitrosalicylic acid (DNS) method [52]. One milliliter of 1% substrate solution (dissolved in 50 mM sodium acetate buffer, pH 5.0), 0.5 mL of *S. sclerotiorum* culture supernatants, and 8.5 mL of substrate solution were incubated at 45 °C for 60 min. After incubation, 1.5 mL DNS was added and re-incubated at 95 °C for 5 min. The absorbance of the reaction mixtures was measured at 540 nm using an ELx800 microplate reader. A standard curve was constructed using galacturonic acid as the standard solution to calculate the activities of PG and PMG. One unit of PG and PMG activities (U) was defined as the amount of enzyme necessary to convert substrate to produce 1 µmol of reducing sugar per minute. All treatments were replicated three times, and this experiment was repeated three times.

### 5.9. Effects of Trichoderma SC5 Fermentation Filtrate on Growth and Infection-Related Genes Expression of S. sclerotiorum

Three 5 mm mycelial discs taken from the edge of a 3-day-old *S. sclerotiorum* colony were transferred to the 250 mL flask containing 90 mL of PDB, with a final concentration of 3% (*v*/*v*) SC5 fermentation filtrate. The same volume of sterile water was used instead of fermentation filtrate as the control. After shaking the culture at 25 °C and 150 rpm for 5 days, the mycelium was collected by filtering through three layers of sterile filter paper. The total RNA of *S. sclerotiorum* was extracted using the E.Z.N.A. Fungal RNA Kit (Omega Bio-Tek, Norcross, GA, USA). The concentration and integrity of RNA were detected by a UV spectrophotometer (Implen NanoPhotometer, Munich, Germany) and 1% agarose gel. The first-strand cDNA was synthesized following the guidelines provided by the first-strand cDNA synthesis kit (Takara Bio-Tek, Dalian, China). Quantitative real-time polymerase chain reaction (qRT-PCR) was employed to assess the expression levels of 14 genes. The functions of the genes selected for qRT-PCR are presented in Table 3. The primers for qRT-PCR were designed with Primer Premier 5 and presented in Table S1. TB GreenTM Premix Ex TaqTM II (Takara Bio, Shiga, Japan) was used for qRT-PCR with a CFX96 Touch Real-time PCR detection system (Bio-Rad, Hercules, CA, USA). Each treatment was set with three technical replicates, and the experiment was performed with three biological replicates. The *β*-*tubulin* [53] and Glyceraldehyde-3-phosphate dehydrogenase (*gpdh*) [54] genes of *S. sclerotiorum* were selected as the internal control. The relative expression level of different genes was calculated using the 2^−∆∆Ct^ method.

### 5.10. Protective and Curative Activities of Trichoderma SC5 Lyophilized Fermentation Filtrate Against S. sclerotiorum on Sunflower Leaves

The protective and curative effects of the *Trichoderma* SC5 lyophilized fermentation filtrate on sunflower leaves were evaluated using the methodology described by Li et al. [68], with slight modifications. The SC5 fermentation filtrate was lyophilized using a freeze-drying instrument (Beijing Songyuan Huaxing Technology Develop Co., Ltd., Beijing, China), and then dissolved with sterile water to make a 50 mg/mL solution. The tested sunflower variety was ‘LD5009’, which is highly susceptible to *S. sclerotiorum*. Sunflower leaves with similar stages, sizes, and growing positions were selected as experimental materials. The selected leaves underwent sterilization using 75% ethanol for 1 min, followed by rinsing with sterile water and blotting drying. The SC5 lyophilized fermentation filtrate underwent dilution with sterile water containing 0.1% Tween 80 to make the final concentrations of 0, 1, 2, 4, 6, and 8 mg/mL.

For the assay of protective activity, the leaves were punctured with sterile needles and sprayed with different concentrations of lyophilized fermentation filtrate until the liquid flowed on the leaf surface. After 24 h of treatment, a 5-mm mycelial disc from 3-day-old *S. sclerotiorum* was inoculated onto the wound site of each leaf. The sunflower leaves were incubated at 25 °C, maintaining 85% relative humidity and a 16 h photoperiod per day. Four days post-inoculation, the lesion diameters were measured and taken on each leaf along two perpendicular axes. The mean of the two perpendicular diameter measurements was computed, and the disease control efficacy (DCE) was determined using the formula: DCE (%) = (d_c_ − d_t_)/d_c_ × 100, where d_c_ denotes the lesion diameter in the water control, and d_t_ represents the lesion diameter in the treated leaves.

In assessing curative activity, *S. sclerotiorum* was first inoculated onto the wound surface of the leaves, and 24 h later, different concentrations of SC5 lyophilized fermentation filtrate were sprayed. The DCE was calculated as described in the protective activity test after inoculation. The experimental design included ten replicates per treatment, with the entire procedure replicated thrice.

### 5.11. Morphological and Molecular Identification of Trichoderma SC5 Strain

The SC5 strain was cultured on PDA plates at 28 °C under a 12/12 h light/dark cycle for 5 days, and the morphological characteristics of the colony, conidia, conidiophores, and phialides of SC5 were observed using a Nikon DS-Ri2 microscope (Nikon, Tokyo, Japan). The size of one hundred conidia was measured by calculating the average size of SC5 conidia. Genomic DNA of SC5 was extracted using the E.Z.N.A.^®^ High Performance (HP) Fungal DNA Kit (OMEGA Bio-Tek, Norcross, GA, USA). The extracted SC5 DNA served as the template for polymerase chain reaction (PCR) based on the amplification of ITS region with ITS1 (5′-TCCGTAGGTGAACCTGCGG-3′) and ITS4 (5′-TCCTCCGCTTATTGATATGC-3′) [69], *EF1-α1* gene with tef71 (5′-CAAAATGGGTAAGGAGGASAAGAC-3′) and tef997 (5′-CAGTACCGGCRGCRATRATSAG-3′) [70], and *RPB*2 gene with *RPB*2-5F (5′-GAYGAYMGWGATCAYTTYGG-3′) and *fRPB*2-7cR (5′-CCCATRGCTTGYTTRCCCAT-3′) [71]. The purified PCR products were sent to Sangon Biotech Company (Shanghai, China). The obtained sequences were compared with those in the National Center for Biotechnology Information (NCBI) (https://blast.ncbi.nlm.nih.gov, accessed on 22 June 2022) using the Basic Local Alignment Search Tool (https://blast.ncbi.nlm.nih.gov/Blast.cgi, accessed on 22 June 2022) to determine their phylogenetic relationships, with accession numbers provided in Table S2. The single-gene datasets were combined into a multi-gene dataset using Sequence Matrix (version 1.7.8) software [72]. The interleaved NEXUS file was formatted utilizing the PAUP* (version 5.0) software. Phylogenetic analysis was conducted with combined genetic regions of ITS, *EF1-α1,* and *RPB*2. Construction of phylogenetic trees from the concatenated sequences was performed using MEGA-X (version 10.1.8) software [73]. The analysis employed the maximum likelihood (ML) method, utilizing Kimura 2 parameter genetic distances (K2), incorporating a gamma distribution (+G) and invariant sites (+I), with 2000 bootstrap replications. Phylogenetic tree editing and visualization were performed using the online tool tvBOT (https://www.chiplot.online/tvbot.html, 29 December 2023) [74].

## Figures and Tables

**Table 1:** Effect of *Trichoderma* spp. fermentation filtrate on the mycelial growth and sclerotial production of *S. sclerotiorum*.

Treatment	Inhibition Rate (%)	Number of Sclerotia Per Plate	Dry Weight of Sclerotia (g)
Water	/	26.7 ± 1.2 a	0.1207 ± 0.0114 a
TN	69.58 ± 3.26 d	0.0 ± 0.0 d	0.0 ± 0.0 c
TS3	83.01 ± 1.28 b	0.0 ± 0.0 d	0.0 ± 0.0 c
SC5	94.65 ± 0.96 a	0.0 ± 0.0 d	0.0 ± 0.0 c
T6	58.99 ± 4.42 e	9.0 ± 1.0 c	0.0577 ± 0.0110 b
P6	74.59 ± 2.26 c	15.3 ± 2.1 b	0.1206 ± 0.0133 a

The inhibition rate (%) was determined at 5 days after inoculation, and the number and dry weight of sclerotia were determined at 20 days after inoculation. The data represent the means ± SD (standard deviation). Different letters in the same column indicate significant differences (*p* < 0.05) in each treatment according to Duncan’s multiple range test.

**Table 2:** Protective and curative activity of *Trichoderma* SC5 fermentation filtrate on sunflower leaves.

Concentrations of Lyophilized Fermentation Filtrates (mg/mL)	Curative Activity		Protective Activity	
	Lesion Diameter (cm)	Control Efficacy (%)	Lesion Diameter (cm)	Control Efficacy (%)
0 (CK)	4.33 ± 0.31 a	/	4.40 ± 0.17 a	/
1	2.68 ± 0.16 b	38.03 ± 3.71 d	2.67 ± 0.15 b	39.39 ± 3.47 e
2	2.15 ± 0.13 c	50.35 ± 3.05 c	1.62 ± 0.24 c	63.26 ± 5.37 d
4	2.06 ± 0.18 c	52.65 ± 4.16 c	1.28 ± 0.23 d	70.83 ± 5.12 c
6	1.68 ± 0.19 d	61.12 ± 4.37 b	0.92 ± 0.13 e	79.17 ± 2.86 b
8	1.06 ± 0.10 e	75.36 ± 2.40 a	0.2 ± 0.10 f	95.45 ± 2.27 a

Values are expressed as means ± SD. Values in each column followed by the different letters are significantly different at the *p* < 0.05 level, according to Duncan’s multiple range test.

**Table 3:** Genes for qRT-PCR analysis and their potential functions in the life cycle and pathogenicity of *S. sclerotiorum*.

Genes Name	Accession Number	Main Functions and/or Putative Mechanisms	References
*Ss-sac1*	SS1G_07715	sclerotia development, virulence	[55]
*SSILT*	SS1G_14133	virulence, suppresses host defense reactions	[56]
*Ss-smk1*	SS1G_11866	sclerotia development	[57]
*Ss-caf1*	SS1G_02486	appressorium formation, sclerotia development	[58]
*Pph1*	SS1G_08489	melanin biosynthesis, infection-cushion production	[59]
*Ss-Sm1*	SS1G_10096	hyphal growth, development of infection-cushions and sclerotia	[60]
*Ss-CVNH*	SS1G_02904	virulence, sclerotia development	[61]
*Ss-Nox1*	SS1G_05661	virulence, sclerotia development	[62]
*Ss-Acp1*	AF221843	encoding acid protease	[63]
*Ss-PG1*	AAM22186	encoding polygalacturonase 1	[64]
*Ss-CutA*	XM_001590986	encoding cutinase	[64]
*Ss-oah1*	SS1G_08218	oxalic acid biosynthesis	[65]
*Ss-pth2*	SS1G_13339	sclerotia development, oxalic acid biosynthesis	[66]
*Ss-sod1*	SS1G_00699	growth, sclerotia development, oxalic acid biosynthesis, virulence	[67]

## Data Availability

Data is contained within the article.

## Supplementary Materials

The following supporting information can be downloaded at https://www.mdpi.com/article/10.3390/ijms26010201/s1.
