# Supplementary material for: Efficacy of Trichoderma longibrachiatum SC5 Fermentation Filtrate in Inhibiting the Sclerotinia sclerotiorum Growth and Development in Sunflower
Source: Int J Mol Sci. 2024 Dec 29;26(1):201. doi: 10.3390/ijms26010201 (PMC11720231; doi:10.3390/ijms26010201)
Supplement: Supplementary file 1 [file ijms-26-00201-s001.zip › ijms-3354706-supplementary.pdf]

Table S1. The primer sequences used for qRT-PCR analysis in this study.

Genes	Sequence (5' to 3')
Ss-sac1	F: GGCGAGTATAACGACAGCGA<br>R: TAATGTCCGTGGTCTGTGGC
SSILT	F: AAGAGCGTAATGGATGGTGG<br>R: AGCAAATGTGGTGCCGACT
Ss-smk1	F: ATCCGATGATCATTGCCAG<br>R: ATCTAGCAAGACCAAAATCGCA
Ss-caf1	F: TTTGGAGCGAAGGGTGGAA<br>R: GGCATCTTATCCAAAGCCGC
Pph1	F: TTCGATTACCTTCCCCTCAC<br>R: AGTCGCACATTGGACCTTCA
Ss-Sm1	F: GGAGTATAAGTGGGAAGGCAAGA<br>R: GAGGGATAGCCCACTGATAACA
Ss-CVNH	F: TCGTACGTCTCTTCCTCCCT<br>R: TGCAGATTCCGCTGTCAACT
Ss-Nox1	F: CGAAAGCCATCGATGAAG<br>R: CGACATCGGCTCCTACAC
Ss-Acp1	F: GCCACCCAAAACGGAGAAT<br>R: GAGGTGAGGACGGAGTTTTGTT
Ss-PG1	F: TCTTGCAGCAGTCGAGAAG<br>R: GTGTTGTGTCCGAGGGAGT
Ss-CutA	F: CTTCGGCGATCCAGATAA<br>R: ATCCATTCCGTATGTTAGATG
Ss-oah1	F: GTGTCGTAGGAACTCCAAAGAA<br>R: CACCAGCCGCAGTATCAATA
Ss-pth2	F: TCCTTTGTAACGGGGAGGGA<br>R: TTCGAGGAAACGTCGTTGGT
Ss-sod1	F: TACATCGCCAATGAAGAAACTG<br>R: GGAGATCCCTCAATCACTGAATAG
β-tubulin	F: TTGGATTTGCTCCTTTGACCAG<br>R: AGCGGCCATCATGTTCTTAGG
gpdh	F: TGGCTCCTACTAAAGTTG<br>R: CAAGCAGTTGGTTGTGCAAG

Table S2. Species, source, and GenBank accession numbers of reference strains used for phylogenetic analysis in this study.

Species	Strain	Locality, Host/Substrate	GenBank Accession		
			ITS	*tef*1	*RPB*2
Trichoderma bannaense	HMAS 248840	China, soil	KY687923	KY688037	KY687979
Trichoderma longibrachiatum	TRS766	Poland, not known	KP009318	KP008884	KP009199
Trichoderma longibrachiatum	Tloum3	China, soil	MT102396	MT081437	MT118251
Trichoderma viride	CBS 119325	Czech Republic	DQ677655	DQ672615	EU711362
Trichoderma koningiopsis	Dis 339c	Ecuador, *Theobroma gileri*	DQ313142	DQ284968	FJ442784
Trichoderma asperellum	GJS 01-294	Saudi Arabia, soil	EU856297	EU856323	FJ150788
Trichoderma asperellum	TRS746	Poland	KP009371	KP008926	KP009073
Trichoderma harzianum	T3	China, soil	KX620136	KX632592	MG917685
Trichoderma andinense	LESF560	Brazil, *Atta cephalotes*	KT278909	KT279038	KT278980
Trichoderma orientale	TRS707	Poland	KP009364	KP008888	KP009202
Trichoderma citrinoviride	DAOM 172792	Canada, wood	EU280098	EU280036	KJ842210
Trichoderma semiorbis	DAOM 167636	Australasian, wood	AY737758	KJ871120	KJ842203
Trichoderma hunua	CBS 238.63	New Zealand, not known	MH858273	KJ665519	KJ665279
Trichoderma virens	DAOM 167652	USA, soil	EU330955	AY750891	AF545547
Trichoderma turrialbense	CBS 112445	Costa Rica,	EU330945	EU338284	EU338321
Trichoderma valdunense	CBS 120923	Austria	FJ860863	FJ860717	FJ860605
Trichoderma velutinum	30.24.06.3	Poland	KP009268	KP008909	KP009180
Trichoderma voglmayrii	CBS 117711	Austria	NR134358	DQ086146	FJ179622
Trichoderma subeffusum	CBS 120929	Austria	FJ860852	FJ860707	FJ860597
Trichoderma tawa	GJS 97-174	Thailand	AY737756	AY737739	AY391956

Trichoderma guizhouense	HGUP0038	China, soil	JN191311	JN215484	JQ901400
Trichoderma gamsii	GJS 04-09	USA, soil	DQ315459	DQ307541	JN133561
Trichoderma taxi	ZJUF0986	China, *Taxus mairei*	DQ470074	DQ859029	DQ859032
Trichoderma trixiae	ATCC32630	Sweden	DQ315445	DQ307526	KC285770*
Protocrea farinosa	CBS 121551	Austria	MH863119	EU703889	EU703935
—	**SC5**	**China, soil**	**ON786721**	**ON808971**	**ON815270**

Notes: *Trichoderma* strain of the present study are marked in bold. The mark '*' means the accession number of another fungal strain from GenBank.
